# Recoverable Palladium-Catalyzed Carbon-Carbon Bond Forming Reactions under Thermomorphic Mode: Stille and Suzuki-Miyaura Reactions

**DOI:** 10.3390/molecules26051414

**Published:** 2021-03-05

**Authors:** Eskedar Tessema, Vijayanath Elakkat, Chiao-Fan Chiu, Zong-Lin Tsai, Ka Long Chan, Chia-Rui Shen, Han-Chang Su, Norman Lu

**Affiliations:** 1 Institute of Organic and Polymeric Materials, National Taipei University of Technology, Taipei 106, Taiwan; eskedartessema18@gmail.com (E.T.); vijayanathe123@gmail.com (V.E.); a66366268@gmail.com (Z.-L.T.); kalongabc@gmail.com (K.L.C.); 2 Department of Pediatrics, Linkou Medical Center, Chang Gung Memorial Hospital, Taoyuan 333, Taiwan; 3 Graduate Institute of Clinical Medical Sciences, College of Medicine, Chang Gung University, Taoyuan 333, Taiwan; 4 Department of Medical Biotechnology and Laboratory Sciences, College of Medicine, Chang Gung University, Taoyuan 333, Taiwan; crshen@mail.cgu.edu.tw; 5 Department of Ophthalmology, Linkou Medical Center, Chang Gung Memorial Hospital, Taoyuan 333, Taiwan; 6 Creditable Service Technology Consultants, New Taipei City 235, Taiwan; henry@mail.creditable.com.tw; 7 Development Center for Smart Textile, National Taipei University of Technology, Taipei 106, Taiwan

**Keywords:** homogeneous, sustainable catalysis, catalyst, fluorous, thermomorphic, recovery, palladium, long-chained, Stille, Suzuki-Miyaura, coupling

## Abstract

The reaction of [PdCl_2_(CH_3_CN)_2_] and bis-4,4′-(R_f_CH_2_OCH_2_)-2,2′-bpy (**1a–d**), where R_f_ = *n*-C_11_F_23_ (**a**), *n*-C_10_F_21_ (**b**), *n*-C_9_F_19_ (**c**) and *n*-C_8_F_17_ (**d**), respectively, in the presence of dichloromethane (CH_2_Cl_2_) resulted in the synthesis of Pd complex, [PdCl_2_[4,4′-bis-(R_f_CH_2_OCH_2_)-2,2′-bpy] (**2a–d**). The Pd-catalyzed Stille arylations of vinyl tributyltin with aryl halides were selected to demonstrate the feasibility of recycling usage with **2a** as the catalyst using NMP (N-methyl-2-pyrrolidone) as the solvent at 120–150 °C. Additionally, recycling and electronic effect studies of **2a–c** were also carried out for Suzuki-Miyaura reaction of phenylboronic acid derivatives, 4-X-C_6_H_4_-B(OH)_2_, (X = H or Ph) with aryl halide, 4-Y-C_6_H_4_-Z, (Y = CN, H or OCH_3_; Z = I or Br) in dimethylformamide (DMF) at 135–150 °C. At the end of each cycle, the product mixtures were cooled to lower temperature (e.g., −10 °C), and then catalysts were recovered by decantation with Pd leaching less than 1%. The products were quantified by gas chromatography/mass spectrometry (GC/MS) analysis or by the isolated yield. The complex **2a**-catalyzed Stille reaction of aryl iodides with vinyl tributyltin have good recycling results for a total of 8 times, with a high yield within short period of time (1–3 h). Similarly, **2a–c**-catalyzed Suzuki-Miyaura reactions also have good recycling results. The electronic effect studies from substituents in both Stille and Suzuki-Miyaura coupling reactions showed that electron withdrawing groups speed up the reaction rate. To our knowledge, this is the first example of recoverable fluorous long-chained Pd-catalyzed Stille reactions under the thermomorphic mode.

## 1. Introduction

Carbon-carbon bond forming reactions are among the most useful and most widely studied synthetic transformations. Most importantly, the Stille, Suzuki-Miyaura, Heck, Negishi and Sonogashira coupling reactions typically catalyzed by palladium have been abundantly used in syntheses and widely studied in recent decades. Stille coupling, which is the palladium-catalyzed cross-coupling of an organostannane and an organohalide, is one of the most powerful methods for the straightforward joining together of carbon-carbon bonds in synthetic chemistry [1,2,3]. It is currently a widely used cross-coupling method for the synthesis of functional molecules and polymers, both in laboratory research and in industry [1,4]. In such kind of coupling reaction, several advantages are observed from the substrate groups. These include the stability and functional group tolerance of stannanes, the broad reaction scope of aryl halides and pseudo-halides, and its chemoselectivity. On the other hand, Suzuki-Miyaura coupling reaction has become a main method in modern synthetic organic chemistry for the preparation of biaryl compounds [5,6]. Therefore, the coupling products of both the reactions have been widely applied in natural products [7,8,9], medicinal chemistry [10,11], chemical biology [12], industrial process [13] and materials [14,15] in a controlled and selective manner.

Homogeneous catalysis of Stille coupling reaction generally takes place in organic solvents and, hence, it is difficult to separate the catalyst from the reaction mixture and recover it at the end of the reaction, leading to wastage of noble metals. Concerns for the environment and scarcity of resources is becoming a challenge, which motivates chemists to look for greener processes that are more economical and ecofriendly. Therefore, developing a catalyst with a better activity, excellent selectivity, easy separation and better yield is everyone’s goal. To solve this problem, several noble strategies involving heterogeneous and improvised homogeneous catalysts have been developed for recycling and reusing Stille catalysts, including the use of Pd complexes supported by recombinant peptide fusion nanoparticles [16], magnetic nanoparticles [17], stainless steel mesh-GO (graphene oxide) nanoparticles [18], polymer [19,20,21], silica [22], porous metal-organic framework (MOF) [23], functionalized nanoporous silica [24,25], bulky ligands [26] and metal nanoparticles [27,28]. Additionally, a few reaction solvent options, including alcohols [29,30], water [31,32,33], and ionic liquid [34,35] were also studied to ensure that successful recovery occurred. Similarly, several recoverable Suzuki-Miyaura catalysts, including the use of Pd complexes supported by agar [36], polymer [37], aminophosphine supported on Al_2_O_3_ [38], perovskite-based [39], and magnetically recoverable ones [40,41] were used.

**Figure 1:**
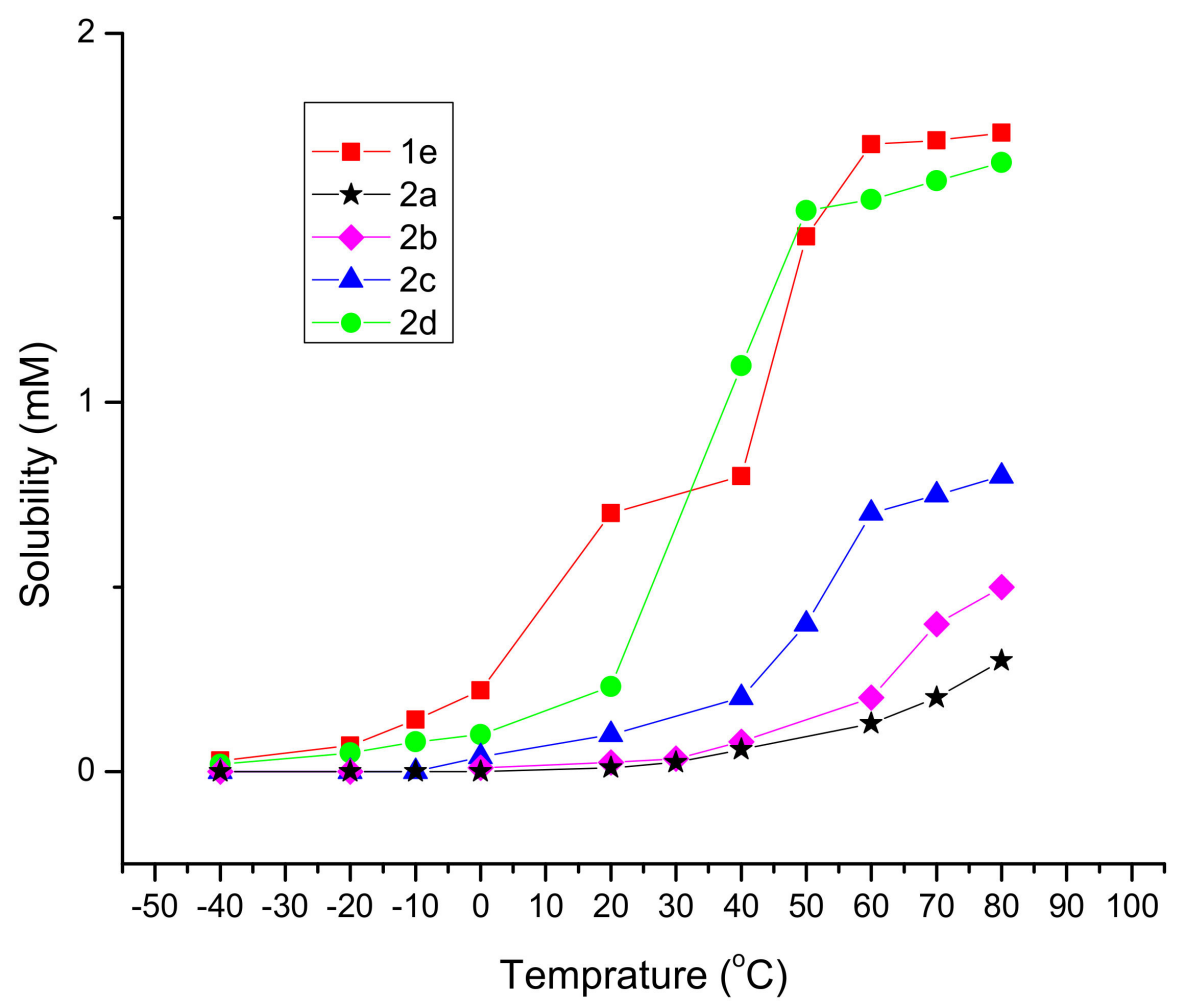
Solubility of Pd complexes **2a–d** and ligand **1e** in dimethylformamide (DMF) as a function of temperature.

In this work, the fluorous long-chained palladium catalysts, [PdCl_2_[4,4′-bis-(n-CF_3_(CF_2_)_10_CH_2_OCH_2_)-2,2′-bpy] (**2a–c**) had been synthesized and been applied to catalyze the Stille and Suzuki-Miyaura reaction of aryl halides with their respective reagents. Recently in Lu’s group, both linear and branched fluorous chains are used to diversify and enrich the fluorous ligand selection pool, when designing the recoverable fluorous catalysis and keeping the enough amount of fluorine content in the complex necessary for this kind of recoverable catalysis. To the best of our knowledge, this is the first example of the application of fluorous long-chained Pd complexes as effective, recoverable catalysts for Stille and Suzuki-Miyaura reactions under thermomorphic condition for many times of re-usage. The thermomorphic property of few fluorous long-chained palladium complexes was previously demonstrated in DMF solvent in Lu’s group and published elsewhere [42,43]. In this study, we have a more complete thermomorphic survey of a series of the chain length of Pd complexes and a related fluorous compound (see Figure 1 below). This type of fluorous long-chained palladium complexes **2a–c** features on homogeneously catalyzing at high temperature in common polar organic solvent and turning to the heterogeneous precipitation at lower temperature. Taking their good catalytic ability and good thermal stability into consideration, these catalysts could be regarded as a very good alternative to homogeneous complexes in sustainable catalysis.

## 2. Results and Discussion

### 2.1. Catalyst Synthesis

**Scheme 1:**
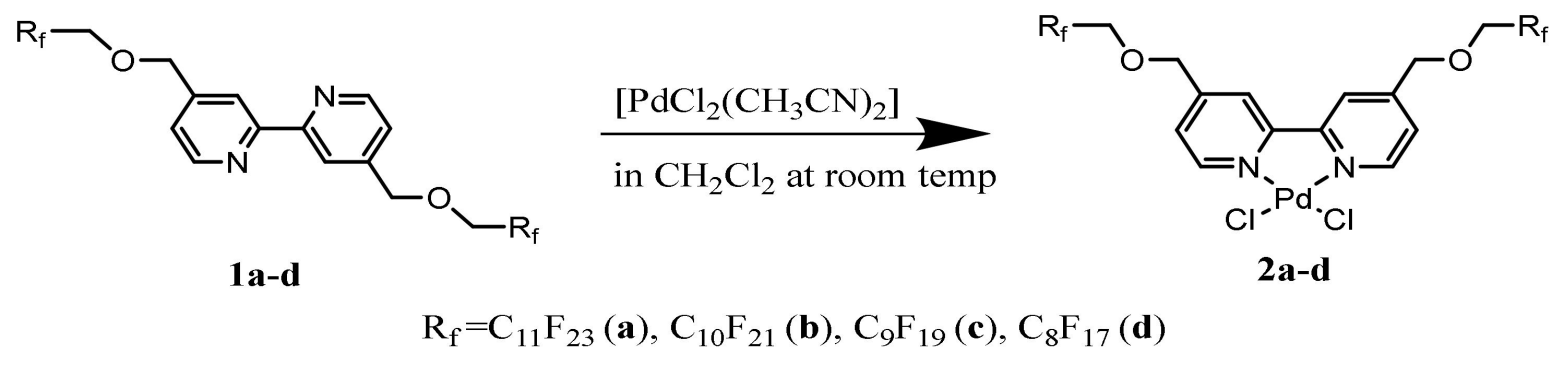
Synthesis of the fluorous long-chained Pd complexes.

The preparation of fluorous long-chained ligands bis-4,4′-(n-R_f_CH_2_OCH_2_)-2,2′-bpy (**1a–e**), where R_f_ = n-C_11_F_23_ (**a**), n-C_10_F_21_ (**b**), n-C_9_F_19_ (**c**), n-C_8_F_17_ (**d**) and n-C_7_F_15_ (**e**), respectively, followed a literature procedure [44,45,46]. The reaction of fluorinated long-chained bipyridine (bpy) ligands, **1a–d**, with [PdCl_2_(CH_3_CN)_2_] in CH_2_Cl_2_, as shown in Scheme 1, resulted in the synthesis of Pd complex [PdCl_2_[4,4′-bis-(R_f_CH_2_OCH_2_)-2,2′-bpy] (**2a–d**) as pale yellow solid already published elsewhere [47,48].

### 2.2. Thermomorphic Property Study

The solubility of Pd complexes **2a–c** in DMF as a function of temperature was shown on the previously published work from Lu’s group [42,43]. As reported here, the solubility was measured by the variable temperature nuclear magnetic resonance (NMR) spectrometer, where the temperatures were varied from −40 to 80 °C (see Figure 1). In this study, we recorded the solubility of Pd complex **2a–d** and a bpy ligand, bis-4,4′-(n-C_7_F_15_CH_2_OCH_2_)-2,2′-bpy (**1e**), by adapting more temperature points when using a similar procedure (see Supplementary Materials). It was shown that compounds **1e** and **2a–d** which showed their solubilities increased dramatically with the increasing temperature. The complex **2d** was soluble in DMF at 20 °C, but complexes **2a–c** were not soluble at −10 °C and below. The Pd complexes **2a–c** were then selected as good candidates for the subsequent catalytic experiments to examine the temperature dependency of the recoverable reactions.

### 2.3. Recoverable Pd Complex-Catalyzed Stille Reaction of Aryl Halides

The fluorous long-chained Pd complex **2a** was then examined for the following Stille reactions where Pd-catalyzed the coupling reaction of vinyl tributyltin (**3**) with aryl iodides (**4–7**) or aryl bromides (**8–11**).

#### 2.3.1. Aryl Iodides

**Scheme 2:**
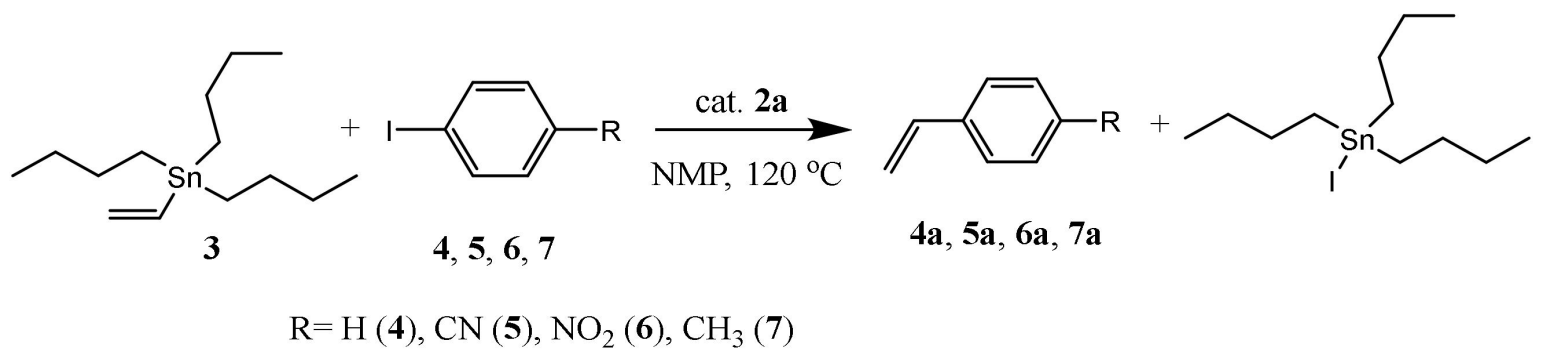
The Pd catalyst (**2a**)-catalyzed Stille reaction of aryl iodides (**4–7**) with vinyl tributyltin (**3**) under the thermomorphic condition.

As shown in Scheme 2, the Pd-catalyzed Stille arylation of vinyl tributyltin (**3**) with aryl iodides was selected to demonstrate the feasibility of recycling usage with **2a** as the catalyst using NMP (N-methyl-2-pyrrolidone) as the solvent under thermomorphic mode, at ca 120 °C for 1–8 h varying in each run for different substituents. At the end of each cycle, the product mixtures were cooled to below −10 °C and centrifuged, and the catalyst was recovered by decantation. The recovered **2a** was added with NMP and substrates to proceed to the next cycle. The products were quantified with GC/MS analysis by comparison to internal standard (anisole). As one example shown in Table 1, **2a**-catalyzed Stille reaction of C_6_H_5_I (**4**) with vinyl tributyltin (**3**) could give rise to the good recycling results for a total of 8 times. To our knowledge, this is the first example of recoverable fluorous Pd-catalyzed Stille reaction under a thermomorphic mode with such a high yield and recyclability.

With electron-withdrawing groups (EWGs) CN and NO_2_ substituents on iodobenzene, the **2a**-catalyzed Stille reactions also gave rise to respective **5a** and **6a** with the excellent yield and recyclability under the thermomorphic mode (see Table 2 and Table 3).

Similar to the recycling observed in the reaction of parent aryl compound (**4**), here complex **2a** could be easily recovered and reused in the Pd-catalyzed Stille arylation of vinyl tributyltin under the thermomorphic mode. The aryl compounds with EWGs exhibited a greater rate (only 1 h as shown in Table 2 and Table 3) than the parent aryl compound, which needed 3–6 h to complete the reaction.

When an electron-releasing group (ERG) CH_3_ substituted on iodobenzene (**7**) was used as a substrate in **2a**-catalyzed Stille reaction, the electron-releasing Me group slowed down the reactions (Table 4), although 8 mol% of catalytic loading was used. Nonetheless, the recyclability could be demonstrated, only with both a longer time and at higher temperature. The yields were 100% throughout, but the temperature was 130 °C for the first five cycles in 8 h and raised to 140 °C time to 9 h in the sixth cycle; it then continued to rise to 150 °C in the seventh and eighth cycles in 10 and 15 h, respectively. 

Sajiki and his co-workers [49] have reported palladium on charcoal-catalyzed ligand-free Stille coupling aryl iodides as shown on Scheme 2. They have used 5 mol% of catalytic loading for 4-cyanoiodobenzne to obtain a yield of 88% in 24 h at an elevated temperature with turnover number (TON) and turnover frequency (TOF) values of 17.6 and 0.73 h^−1^, respectively. For same catalytic loading and duration of time, they have obtained 63% yield at 50 °C by using 4-nitroiodobenzene with TON and TOF values of 12.6 and 0.52 h^−1^, respectively. However, for a similar reaction only 1 mol% of catalytic loading was needed for our catalyst **2a** to have a 100% yield at 120 °C in 1 h with TON and TOF values of 100 and 100, respectively. Thus, the activity of catalyst **2a** reported here is robust with better recovery of 8 cycles.

Thus, the iodobenzene bearing an electron-withdrawing group (CN and NO_2_) smoothly underwent the cross-coupling reaction compared to the non-substituted (R = H) case or the case bearing an electron-releasing group (R = CH_3_) to give the desired vinyl derivatives in excellent yields.

#### 2.3.2. Aryl Bromides

**Scheme 3:**
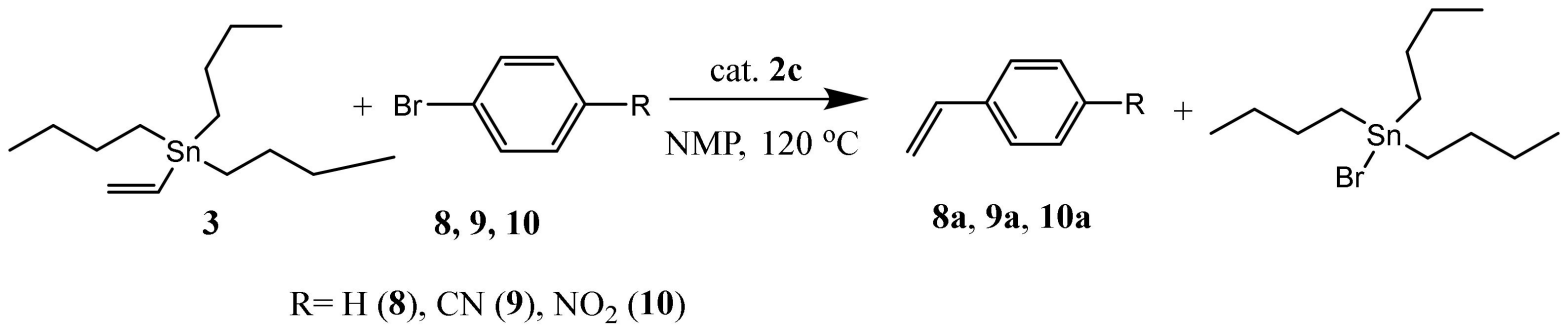
The Pd catalyst (**2a**)-catalyzed Stille reaction of aryl bromides (**8–10**) with vinyl tributyltin (**3**) under the thermomorphic condition.

The Pd complex **2a** was also found to be effective in the Pd-catalyzed Stille vinylation of less reactive aryl bromide under thermomorphic mode, as shown in Scheme 3. Table 5 shows the results of Stille reaction of the unsubstituted aryl bromides (**8**) with vinyl tributyltin (**3**) using NMP as a solvent.

Table 6 and Table 7 show the good results of reactivities and recycling of **2a**-catalyzed Stille reaction of the EWGs CN and NO_2_-containing aryl bromides (**9** and **10**, respectively) with vinyl tributyltin (**3**) using NMP as a solvent. The same reaction had been performed by using an aryl bromide containing the ERG CH_3_ in the same conditions expressed above. Although the reaction proceeded forming the products, it took a very long time to finish the reaction. Thus, those data have not been presented here.

Lerebours and Wolf [50] have reported that they have used 6 mol% of Pd-catalyst at 140 °C for 24 h to catalyze a similar kind of Stille reaction (Scheme 3) which uses bromobenzene as a substrate. As a result, they have obtained a product in 95–96% yield with a TOF = 0.67 h^−1^, after the first two cycles of reactions, but it was found that the catalytic performance of the recovered Pd catalyst slightly diminished after each step to the 4th cycle. However, at similar temperature we have obtained a better yield in 12 h (for the first two cycles) by using unsubstituted aryl bromide substrate with a TOF = 0.83 h^−1^. Thus, a catalytic efficiency of **2a** was proved to be better comparatively. Additionally, for the EWG substituted aryl bromides, only 6 h was required to reach a yield of 100% in the first 5 cycles, even at a slightly reduced temperature (120 °C; see Table 7). Additionally, catalyst **2a** can be recovered and reused for up to 8 cycles by keeping their activity used for all kinds of substrates.

#### 2.3.3. Purification of Crude Product Mixture from Pd-Catalyzed Stille Reaction

**Scheme 4:**
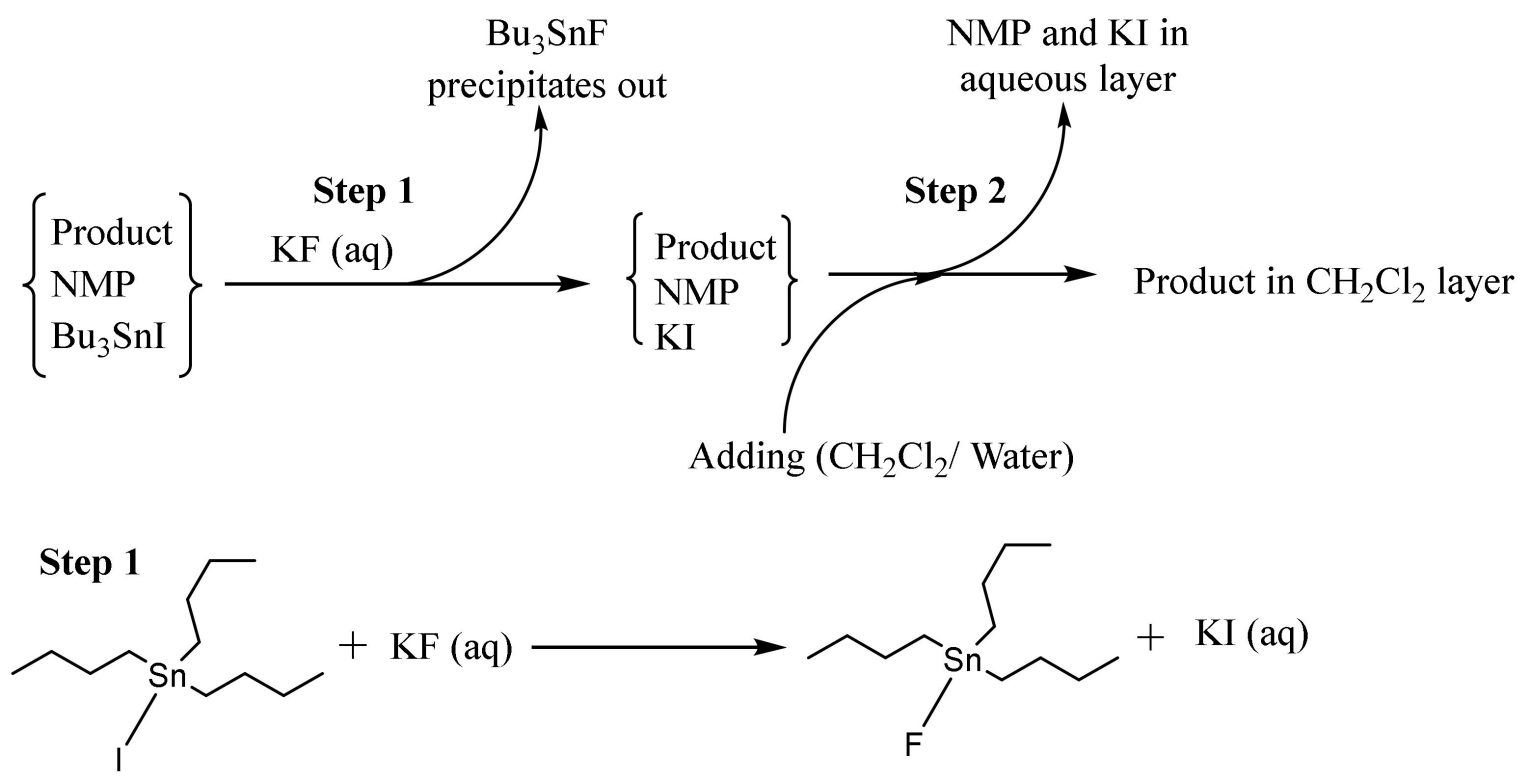
Schematic drawing of the purification process of the Stille reaction product mixture.

At the end of the reaction, a two-step procedure was followed to obtain a pure product. On the first step, the crude reaction mixture obtained was added to an aqueous solution of potassium fluoride (KF) according to a reported literature procedure using an alcoholic KF solution [51]. The KF solution was allowed to react with the tin by-product in the mixture, so that the tin by-product precipitates into a white powder of polymeric Bu_3_SnF [2] and is removed by filtration. This was repeated until no residue was observed in the flask. Finally, water and CH_2_Cl_2_ were used for extraction in the second step, so that the solvent (NMP) would be trapped into the water layer with the KI after several extractions to achieve the pure product in the CH_2_Cl_2_ layer (Scheme 4). Therefore, the recoverability of Pd catalyst and easy isolation of product demonstrated that **2a**-catalyzed Stille reactions of aryl halides are good examples of sustainable catalysis.

### 2.4. Recoverable Pd Complex-Catalyzed Suzuki-Miyaura Reaction of Aryl Halides

#### 2.4.1. Aryl Iododes

**Scheme 5:**
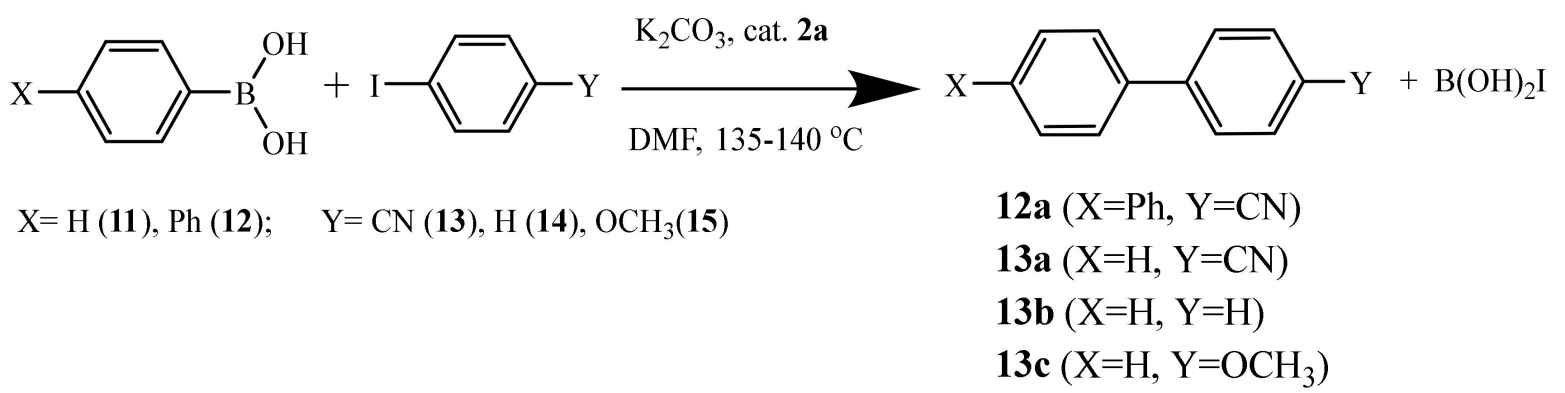
The Pd catalyst (**2a**)-catalyzed Suzuki-Miyaura reaction of two phenyl boronic acid derivatives with three iodobenzene derivatives under the thermomorphic condition.

We also evaluated complex **2a** in Suzuki-Miyaura reaction of aryl iodides (**13**–**15**) with phenyl boronic acid derivatives (**11**, **12**) as indicated in Scheme 5. The results showed that **2a** could effectively catalyze the Suzuki-Miyaura reactions as shown in Table 8. The catalyst could be recycled and reused several times. The electron withdrawing phenyl substituent on the 4-biphenylboronic acid (**12**) was seen to speed up the reaction (see entries 1–3 in Table 8). It is known that the electron-withdrawing CN substituent on aryl iodide (**14**) could speed up Suzuki-Miyaura reactions. Thus, the entries 1–6 in Table 8 were tested first for **2a**-catalyzed Suzuki-Miyaura reaction of 4-cyanoiodobenzene (when Y = CN) (**14**). After the 1st run, the recovered catalyst was subjected to the normal washing steps and then reused for the next runs. For two sets (entries 1–3 and entries 4–6) of three runs, the reactions were all completed with high yields within 4 h at 135 and 140 °C for 4-biphenyl boronic acid (**12**) and phenyl boronic acid (**11**), respectively.

For entries 7 and 8 of Table 8, iodobenzene (when Y = H) (**14**) with phenyl boronic acid (**11**) were used as the substrates for the **2a**-catalyzed Suzuki-Miyaura reaction. The results demonstrated that complex **2a**, was a good recoverable catalyst for the two runs with the respective yields of 80% and 75% under the conditions outlined in Scheme 5. For entries 9 and 10, these two results also showed that **2a**-catalyzed Suzuki-Miyaura reactions were efficient and recyclable. Although these two Suzuki-Miyaura reactions using the less reactive 4-methoxyiodobenzene (when Y = OCH_3_) (**15**) were found to be slower with the reduced yields of 70% and 60% within 4 h for entries 9 and 10, respectively. Thus, the results in Table 8 have clearly demonstrated that the fluorous long-chained Pd complex (**2a**) is an excellent thermomorphic catalyst for C–C forming Suzuki-Miyaura reactions of aryl iodides.

#### 2.4.2. Aryl Bromides

The catalytic recovery of fluorous long-chained Pd complexes **2a–c** were then examined for the following Suzuki-Miyaura reaction experiment to catalyze the coupling of phenyl boronic acid (**11**) with 4-cyanobromobenzene (**16**) under the thermomorphic condition.

**Scheme 6:**
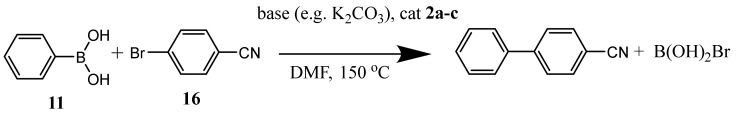
The Pd catalyst (**2a–c**)-catalyzed Suzuki-Miyaura reaction of phenyl boronic acid (**11**) with 4-cyanobromobenzene (**12**) under the thermomorphic condition.

The Pd-catalyzed Suzuki-Miyaura reactions of phenyl boronic acid (**11**) with 4-cyanobromobenzene (**12**) (see Scheme 6) were successfully carried out under the thermomorphic condition at a 3 mol% of catalytic loading. It was shown that, at an elevated temperature a higher yield was obtained within 8 h. The catalytic recovery has was proven to be robust for 8, 8 and 9 consecutive cycles for **2a**, **2b** and **2c**, respectively. Their good results are shown in Table 9, Table 10 and Table 11, respectively.

Note: after 8 cycles, the recovered Pd catalyst (**2b**) was checked by the ^19^F NMR method. The results show that the ^19^F NMR spectrum of catalyst **2b** are exactly the same before and after (8 cycles) reactions (see Figure S1 in Supplementary Materials).

### 2.5. Detection of Metal Recovery by ICP-MS

The amount of Pd leaching of catalyst (**2a**) in the product solution of Stille reaction after centrifugation from the two randomly selected specific runs from each table were determined by inductively coupled plasma mass spectrometry (ICP-MS). The results indicate that less than 1% of the Pd was leached out to the solution from the catalyst as shown in Table 12 and Table 13. Most of the recovery studies in Stille reaction reported so far have not included ICP-MS data in their works.

Similarly, a Pd leaching study was done for two randomly selected specific runs from each table of Suzuki-Miyaura reactions (see Table 14). From these tables, we can find that the recovery rate of the fluorous long-chained palladium, catalysts **2a–c** can reach up to 99%, under the thermomorphic system. Almost all the metal catalysts could be recycled for the next reaction and get reused. The negligible Pd loss during the 8 cycles did not seem to affect the catalytic activity much, which proves that metal catalysts **2a–c** can achieve the goals of sustainable catalysis.

## 3. Experimental

### 3.1. General Procedures

Gas chromatographic/mass spectrometric data were obtained using an Agilent 6890 Series gas chromatograph (Taipei, Taiwan) with a series 5973 mass selective detector. Reactions were monitored with a HP 6890 GC using a 30 m 0.250 mm HP-1 capillary column with a 0.25 mm stationary phase film thickness. The flow rate was 1 mL/min and split-less. Fourier transform infrared (FT-IR) spectra were obtained on a PerkinElmer RX I FT-IR spectrometer (Taipei, Taiwan). NMR spectra were recorded on Bruker AM 500 and 300 spectrometers (Taipei, Taiwan) using 5 mm o.d. sample tubes.

### 3.2. Starting Materials

The chemicals, reagents and solvents employed were commercially available and used as received. R_f_CH_2_OH where R_f_ = n-C_11_F_23_ (**a**), n-C_10_F_21_ (**b**), n-C_9_F_19_ (**c**) and n-C_8_F_17_ (**d**), and C_87_F_15_ (**e**) were purchased from either Aldrich or SynQuest (Taipei, Taiwan).

### 3.3. Preparation of Palladium Complexes

The fluorous bipyridyl ligandes were prepared using the literature method [42]. The reaction of [PdCl_2_(CH_3_CN)_2_] with fluorinated bipyridine derivatives, bis-4,4′-(n- R_f_CH_2_OCH_2_)-2,2′-bpy (**1a–d**), resulted in the synthesis of [PdCl_2_[4,4′-bis-(n-CF_3_(CF_2_)_10_CH_2_OCH_2_)-2,2′-bpy] (**2a–d**) as pale yellow solids, where R_f_ = n-C_11_F_23_ (**a**), n-C_10_F_21_ (**b**), n-C_9_F_19_ (**c**) and n-C_8_F_17_ (**d**), respectively [44,45,46,47,48,52,53,54,55,56,57]. The reaction was stirred in CH_2_Cl_2_ under nitrogen for 6 h, and the complexes **2a–d** would then precipitate from the reaction mixture. The precipitate was collected after removing the solvent by using vacuum pump and finally washed with methanol to remove the excess metal. The yield was about 85%.

### 3.4. Analytical Data for the Ligands and Pd Metal Complexes

Analytical data of ligand **1e** (as a representative example; for others, see refs [44,45,46,47,48]). Yield: 90%. m.p.: 93–96 °C. FT-IR (cm^−1^): v (bpy, m) 1602, 1561; v (CF_2_ stretch, s) 1242, 1208, 1147. ^1^H-NMR (500 MHz, DMSO-d_6_, rt): δ (ppm) 8.67 (d, *J* = 5.0 Hz, 2H, H6), 8.34 (s, 2H, H3), 7.34 (d, *J* = 5.0 Hz, 2H, H5), 4.77 (s, 4H, bpy-CH_2_), 4.04 (t, *J* = 14.6 Hz, 4H, -CH_2_CF_2_-); ^13^C-NMR (113 MHz, CDCl_3_, 350 K): δ (ppm) 155.9, 149.5, 146.8, 121.8, 119.1 (10C, bpy), 118–108 (-C_7_F_15_), 72.9 (bpy-CH_2_O-), 67.6 (-CH_2_CF_2_-); ^19^F-NMR (470 MHz, CDCl_3_, 350 K): δ (ppm) −80.8 (t, *J* = 9.7 Hz, 6F, -CF_2_CF_3_), −119.3 (t, *J* = 8.6 Hz, 4F, -CH_2_CF_2_), −122.0 (8F), −122.7 (4F), −123.2 (4F), −126.1 (4F). GC/MS: 582 (M^+^-OCH_2_C_7_F_15_), 183 (M^+^-2 (OCH_2_C_7_F_15_)), 91 (C_6_H_5_N^+^).

For Analytical data of complexes **2a–d**, see the literature methods in [42,43,44].

### 3.5. Procedures in Catalytic Stille Reaction and Recovery

In a typical run, an aryl iodide (1 mmol) and vinyl tributyltin (1.02 mmol) were charged into a 10 mL reaction tube containing a magnetic stirrer bar, then the Pd catalyst (1 mol%) and followed by NMP solvent (5 mL). Then the reaction mixture was set to react at 120 °C for the given period of time before GC/MS analysis was done to confirm the completion of the reaction. Once the catalytic recovery was done, the pure product was isolated by using CH_2_Cl_2_/H_2_O extraction and the CH_2_Cl_2_ layer was pumped under reduced pressure to obtain the pure product. The product was finally analyzed by using ^1^H-NMR spectroscopy.

The Pd-catalyzed Stille reactions of all the aryl halide substrates with **3** were carried out under the thermomorphic condition that effectively confirmed the practicability of reutilizing the catalyst in NMP solvent. For every round, the catalytic reaction was performed at 120–150 °C under N_2_ gas. At the end of each run, the product mixtures were put into a freezer (−10 °C) for about five minutes and then centrifuged to separate the catalyst and reaction mixture. After the catalyst was recovered by decantation and washed three times by the same solvent, it was again supplied with the same amounts of NMP solvent, aryl halide and vinyl tributyltin to continue to the next round. The products were tracked with GC/MS (see Section 3 in Supplementary Materials) and ^1^H NMR, using anisole as an internal standard until the completion of the reaction was confirmed.

### 3.6. Procedures in Catalytic Suzuki-Miyaura Reaction and Recovery

In a typical run, 4-cyanoiodobenzene (0.3 mmol) and phenylboronic acid (0.2 mmol) were charged into 10 mL reaction tube containing a magnetic stirrer bar, then the Pd catalyst (3 mol%) and followed by DMF solvent (5 mL). Then the reaction mixture was set to react at 150 °C for 8 h before GC/MS analysis was done to confirm the completion of the reaction. The Pd-catalyzed Suzuki-Miyaura reactions of boronic acid substrates with aryl halides were carried out under the thermomorphic condition that effectively confirmed the practicability of reutilizing the catalyst in a DMF solvent. Every round the catalytic reaction was performed at 135, 140 or 150 °C under N_2_ gas. At the end of each run, the product mixtures were put into a freezer (−10 °C) for about 2 h and then centrifuged to separate the catalyst and reaction mixture. After the catalyst was recovered by decantation and washed three times, it was again supplied with the same amounts of DMF solvent and the substrates to continue to the next round. The products were tracked with GC/MS (see Section 3 in Supplementary Materials) and ^1^H NMR.

## 4. Conclusions

The recovery and recycling of catalysts in Pd-catalyzed Stille and Suzuki-Miyaura coupling reactions employing the thermomorphic condition were successfully demonstrated. The prepared Pd complexes **2a–c** had very high fluorine contents and worked well in both Stille and Suzuki-Miyaura reactions up to 8 cycles. The product yield was also higher in most reaction runs; sometimes it even reached 100%. In the Stille reaction by using catalyst **2a**, when aryl iodides were used as the substartes, good catalytic activities and recoverability were observed for all the cases while the R group was unsubstituted, EWG or ERG. The electronic effects from the R group could also be easily observed with R = EWG speeding up the reaction and R = ERG slowing down the reaction. The metal leaching study for the Stille reaction via the ICP-MS method showed that the Pd metal in the complex was effectively recovered throughout the catalytic cycles with almost no loss in activity. Similarly, the **2a**-catalyzed Suzuki-Miyaura reaction of iodobenzene derivatives with biphenyl boronic acid derivatives took place with good catalytic activities and recoverability. Additionally, in the extended work, the metal complexes **2a–c** were also shown to be active in Pd-catalyzed Suzuki-Miyaura reactions of 4-cyanobromobenzene with phenyl boronic acid. Previously, we have reported that this type of fluorous long-chained Pd complexes have good catalytic activity for Heck and Sonogashira reactions under the thermorphic mode. Therefore, we can conclude that the catalysts **2a–c** are effective in all major Pd-catalyzed C-C coupling reactions for the sustainable research.

## Figures and Tables

**Table 1:** Recycling results of **2a**-catalyzed Stille reaction of iodobenzene (**4**) and vinyl tributyltin (**3**) under the thermomorphic mode.

Cycle No.	Time (h)	Temp (°C)	Yield (%) ^a^	TON ^c^
**1**	3	120	100 (99) ^b^	20
**2**	3	120	100 (91)	20
**3**	3	120	100 (95)	20
**4**	3	120	100	20
**5**	4	130	100	20
**6**	4	130	100	20
**7**	6	130	100	20
**8**	6	130	100	20

Reaction conditions: Temp (T) = 120–130 °C, cat. **2a** (5 mol%), **4** (204 mg, 1 mmol), **3** (333 mg, 1.05 mmol), solvent: 5 mL. ^a^: measured by GC/MS (see Supplementary Materials); ^b^: isolated yield in parenthesis; ^c^: turnover number.

**Table 2:** Recycling results of **2a**-catalyzed Stille reaction of 4-cyanoiodobenzene (**5**) and vinyl tributyltin (**3**) under the thermomorphic mode.

Cycle No.	Time (h)	Temp (°C)	Yield (%) ^a^	TON
**1**	1	120	100 (98) ^b^	100
**2**	1	120	100	100
**3**	1	120	100 (95)	100
**4**	1	120	100	100
**5**	1	120	100 (99)	100
**6**	1	120	100	100
**7**	1	120	99	99
**8**	1	120	98	98

Reaction conditions: T = 120 °C, cat. **2a** (1 mol%), **5** (229 mg, 1 mmol), **3** (320.3 mg, 1.01 mmol), solvent: 5 mL ^a^: measured by GC/MS; ^b^: isolated yield in parenthesis.

**Table 3:** Recycling results of **2a**-catalyzed Stille reaction of 4-nitroiodobenzene (**6**) and vinyl tributyltin (**3**) under the thermomorphic mode.

Cycle No.	Time (h)	Temp (°C)	Yield (%) ^a^	TON
**1**	1	120	100 (98) ^b^	100
**2**	1	120	100 (95)	100
**3**	1	120	100 (97)	100
**4**	1	120	100	100
**5**	1	120	100	100
**6**	1	120	100	100
**7**	1	120	99	99
**8**	1	120	97	97

Reaction conditions: T = 120 °C, cat. **2a** (1 mol%), **6** (249 mg, 1 mmol), **3** (320.3 mg, 1.01 mmol), solvent: 5 mL ^a^: measured by GC/MS; ^b^: isolated yield in parenthesis.

**Table 4:** Recycling results of **2a**-catalyzed Stille reaction of 4-iodotoluene (**7**) and vinyl tributyltin (**3**) under thermomorphic mode.

Cycle No.	Time (h)	Temp (°C)	Yield (%) ^a^	TON
**1**	8	130	100 (98) ^b^	12.5
**2**	8	130	100 (95)	12.5
**3**	8	130	100 (99)	12.5
**4**	8	130	100	12.5
**5**	8	130	100	12.5
**6**	9	140	100	12.5
**7**	10	150	100	12.5
**8**	15	150	100	12.5

Reaction conditions: T = 130–150 °C, cat. **2a** (8 mol%), **7** (218 mg, 1 mmol), **3** (323.4 mg, 1.02 mmol), solvent: 5 mL ^a^: measured by GC/MS; ^b^: isolated yield in parenthesis.

**Table 5:** Recycling result of **2a**-catalyzed Stille reaction of bromobenzene (**8**) and vinyl tributyltin (**3**) under the thermomorphic mode.

Cycle No.	Time (h)	Temp (°C)	Yield (%) ^a^	TON
**1**	12	140	100	10
**2**	12	140	100 (99) ^b^	10
**3**	12	140	100	10
**4**	14	150	100	10
**5**	14	150	100	10
**6**	20	150	100 (97)	10
**7**	24	150	95	9.5
**8**	30	150	86	8.6

Reaction conditions: T = 140–150 °C, cat. **2a** (10 mol%), **8** (157 mg, 1 mmol), **3** (475.6 mg, 1.5 mmol), solvent: 5 mL ^a^: measured by GC/MS; ^b^: isolated yield in parenthesis.

**Table 6:** Recycling results of **2a**-catalyzed Stille reaction of 4-cyanobromobenzene (**9**) and vinyl tributyltin (**3**) under the thermomorphic mode.

Cycle No.	Time (h)	Temp (°C)	Yield (%) ^a^	TON
**1**	4	140	100	10
**2**	5	140	100 (98) ^b^	10
**3**	5	140	100	10
**4**	6	140	100 (98)	10
**5**	6	140	100	10
**6**	7	140	100 (97)	10
**7**	10	140	100	10
**8**	18	140	100	10

Reaction conditions: T = 140 °C, cat. **2a** (10 mol%), **9** (182 mg, 1 mmol), **3** (475.6 mg, 1.5 mmol), solvent: 5 mL ^a^: measured by GC/MS; ^b^: isolated yield in parenthesis.

**Table 7:** Recycling results of **2a**-catalyzed Stille reaction of 4-nitrobromobenzene (**10**) and vinyl tributyltin (**3**) under the thermomorphic mode.

Cycle No.	Time (h)	Temp (°C)	Yield (%) ^a^	TON
**1**	4	120	100	10
**2**	4	120	100	10
**3**	4	120	100 (98) ^b^	10
**4**	4	120	100	10
**5**	4	120	100 (98)	10
**6**	5	150	100	10
**7**	15	150	100	10
**8**	20	150	100	10

Reaction conditions: T = 120–150 °C, cat. **2a** (10 mol%), **10** (202 mg, 1 mmol), **3** (475.6 mg, 1.5 mmol), solvent: 5 mL ^a^: measured by GC/MS; ^b^: isolated yield in parenthesis.

**Table 8:** Recycling results of **2a**-catalyzed Suzuki-Miyaura reaction of two phenyl boronic acid derivatives, 4-X-C_6_H_4_-B(OH)_2_ [X = H (**11**) or Ph (**12**)], with three iodobenzene derivatives, 4-Y-C_6_H_4_-I, [Y = CN (**13**), H (**14**) or OCH_3_(**15**)] under the thermomorphic condition.

Entry	Cycle	Reactant A (X=)	Reactant B (Y=)	Temp (°C)	Yield (%) ^a^	TON
**1**	1st	Ph	CN	135	100	33.3
**2**	2nd	Ph	CN	135	100	33.3
**3**	3rd	Ph	CN	135	100	33.3
**4**	1st	H	CN	140	95	31.7
**5**	2nd	H	CN	140	92	30.7
**6**	3rd	H	CN	140	85	28.3
**7**	1st	H	H	140	80	26.7
**8**	2nd	H	H	140	75	25
**9**	1st	H	OCH_3_	140	70	23.3
**10**	2nd	H	OCH_3_	140	60	20

Reaction conditions: Time = 4 h (for all entries), T = 135 or 140 °C, cat. **2a** (3 mol%), **11** or **12** (0.2 mmol), **13**, **14** or **15** (0.3 mmol), solvent: 5 mL. ^a^: measured by GC/MS.

**Table 9:** Recycling results of **2a**-catalyzed Suzuki-Miyaura reaction of aryl boronic acid (**11**) with 4-cyanobromobenzene (**16**) under the thermomorphic mode.

Cycle No.	Time (h)	Temp (°C)	Yield (%) ^a^	TON
**1**	8	150	99	33
**2**	8	150	99	33
**3**	8	150	99 (98) ^b^	33
**4**	8	150	92	30.7
**5**	8	150	81	27
**6**	8	150	78 (77)	26
**7**	8	150	73	24.3
**8**	8	150	78	26

Reaction conditions: T = 150 °C, cat. **2a** (3 mol%), **11** (202 mg, 1 mmol), **16** (475.6 mg, 1.5 mmol), solvent: 5 mL. ^a^: measured by GC/MS. ^b^: isolated yield in parenthesis.

**Table 10:** Recycling results of **2b**-catalyzed Suzuki-Miyaura reaction of aryl boronic acid (**11**) with 4-cyanobromobenzene (**16**) under the thermomorphic mode.

Cycle No.	Time (h)	Temp (°C)	Yield (%) ^a^	TON
**1**	8	150	99	33
**2**	8	150	97	32.3
**3**	8	150	99 (97) ^b^	33
**4**	8	150	99	33
**5**	8	150	99	33
**6**	8	150	99	33
**7**	8	150	99	33
**8**	8	150	92	30.7

Reaction conditions: T = 150 °C, cat. **2b** (3 mol%), **11** (202 mg, 1 mmol), **16** (475.6 mg, 1.5 mmol), solvent: 5 mL. ^a^: measured by GC/MS. ^b^: isolated yield in parenthesis.

**Table 11:** Recycling results of **2c**-catalyzed Suzuki-Miyaura reaction of aryl boronic acid (**11**) with 4-cyanobromobenzene (**16**) under the thermomorphic mode.

Cycle No.	Time (h)	Temp (°C)	Yield (%) ^a^	TON
**1**	8	150	99	33
**2**	8	150	98	32.7
**3**	8	150	99 (98) ^b^	33
**4**	8	150	99	33
**5**	8	150	98	32.7
**6**	8	150	99	33
**7**	8	150	99	33
**8**	8	150	92 (89)	30.7
**9**	8	150	83	27.7

Reaction conditions: T = 150 °C, cat. **2c** (3 mol%), **11** (40 mg, 0.2 mmol), **16** (36.6 mg, 1.5 mmol), solvent: 5 mL. ^a^: measured by GC/MS. ^b^: isolated yield in parenthesis.

**Table 12:** Pd leaching (%) after **2a**-catalyzed Stille coupling of aryl iodides from inductively coupled plasma mass spectrometry (ICP-MS).

Table No.-Cycle No.	Pd Detected from ICP-MS (In ppm) ^a^	Wt of the Catalyst Used (mg)	Pd Leaching (%)	Pd Recovery (%)
**1–1**	2.932	80	0.11	99.89
**1–8**	5.953	80	0.21	99.79
**2–1**	4.109	16	0.72	99.28
**2–5**	1.372	16	0.27	99.73
**3–2**	0.37	16	0.06	99.94
**3–6**	0.393	16	0.08	99.92
**4–3**	2.673	128	0.07	99.93
**4–7**	21.86	128	0.46	99.54

^a^: Pd (in ppm) detected from ICP-MS analysis.

**Table 13:** Pd leaching (%) after **2a**-catalyzed Stille coupling of aryl bromides from ICP-MS.

Table No.-Cycle No.	Pd Detected from ICP-MS (In ppm) ^a^	Wt of the Catalyst Used (mg)	Pd Leaching (%)	Pd Recovery (%)
**5–1**	2.514	160	0.05	99.95
**5–3**	4.398	160	0.08	99.92
**6–2**	34.89	160	1.18	98.82
**6–4**	15.08	160	0.63	99.37
**7–5**	0.62	160	0.03	99.97
**7–8**	21.76	160	0.76	99.24

^a^: Pd (in ppm) detected from ICP-MS analysis.

**Table 14:** Pd leaching (%) after **2a–c**-catalyzed Suzuki-Miyaura coupling of aryl bromides from ICP-MS.

Table No.-Cycle No.	Pd Detected from ICP-MS (In ppm) ^a^	Wt of the Catalyst Used (mg)	Pd Leaching (%)	Pd Recovery (%)
**8–1**	5.12	47	0.40	99.6
**8–3**	7.69	47	0.60	99.4
**9–1**	4.08	44	0.31	99.69
**9–5**	12.32	44	0.99	99.01
**10–3**	3.33	41	0.26	99.74
**10–5**	13.03	41	1.06	98.94

^a^: Pd (in ppm) detected from ICP-MS analysis.

## Data Availability

The data presented in this study are available in the article.

## Supplementary Materials

The materials are available online. They include thermomorphic curve studies, GC/MS data of products, and Figure S1. ^19^F NMR spectrum of the catalyst **2b**.

## Sample Availability

Some of the fluorous palladium complexes are available from the authors.

## References

[1] Cordovilla C., Bartolomé C., Martínez-Ilarduya J.S.M., Espinet P. (2015). The Stille reaction, 38 years later. ACS Catal..

[2] Lee V. (2019). Application of copper (i) salt and fluoride promoted Stille coupling reactions in the synthesis of bioactive molecules. Org. Biomol. Chem..

[3] Nikoorazm M., Ghorbani-Choghamarani A., Khanmoradi M. (2016). Application of Pd-2A3HP-MCM-41 to the Suzuki, Heck and Stille coupling reactions and synthesis of 5-substituted 1H-tetrazoles. Appl. Organomet. Chem..

[4] Wang D.-Y., Kawahata M., Yang Z.-K., Miyamoto K., Komagawa S., Yamaguchi K., Wang C., Uchiyama M. (2016). Stille coupling via C–N bond cleavage. Nat. Commun..

[5] Miyaura N., Suzuki A. (1995). Palladium-catalyzed cross-coupling reactions of organoboron compounds. Chem. Rev..

[6] Suzuki A.J. (1999). Recent advances in the cross-coupling reactions of organoboron derivatives with organic electrophiles, 1995–1998. J. Organomet. Chem..

[7] Sofiyev V., Navarro G., Trauner D. (2008). Biomimetic synthesis of the shimalactones. Org. Lett..

[8] Souris C., Frébault F., Patel A., Audisio D., Houk K.N., Maulide N. (2013). Stereoselective synthesis of dienyl-carboxylate building blocks: Formal synthesis of inthomycin C. Org. Lett..

[9] Heravi M.M., Hashemi E., Azimian F. (2014). Recent developments of the Stille reaction as a revolutionized method in total synthesis. Tetrahedron.

[10] Lord A.M., Mahon M.F., Lloyd M.D., Threadgill M.D. (2009). Design, synthesis, and evaluation in vitro of quinoline-8-carboxamides, a new class of poly (adenosine-diphosphate-ribose) polymerase-1 (PARP-1) inhibitor. J. Med. Chem..

[11] Cullen M.D., Deng B.L., Hartman T.L., Watson K.M., Buckheit R.W., Pannecouque C., Cushman M. (2007). Synthesis and biological evaluation of alkenyldiarylmethane HIV-1 non-nucleoside reverse transcriptase inhibitors that possess increased hydrolytic stability. J. Med. Chem..

[12] Nicolaou K.C., King N.P., Finlay M.R.V., He Y., Roschangar F., Vourloumis D., Hepworth D. (1999). Total synthesis of epothilone E and related side-chain modified analogues via a Stille coupling based strategy. Bioorg. Med. Chem..

[13] Johansson Seechurn C.C., Kitching M.O., Colacot T.J., Snieckus V. (2012). Palladium-catalyzed cross-coupling: A historical contextual perspective to the 2010 Nobel Prize. Angew. Chem. Int. Ed..

[14] Carsten B., He F., Son H.J., Xu T., Yu L. (2011). Stille polycondensation for synthesis of functional materials. Chem. Rev..

[15] Choudary B.M., Madhi S., Chowdari N.S., Kantam M.L., Sreedhar B. (2002). Layered double hydroxide supported nanopalladium catalyst for Heck-, Suzuki-, Sonogashira-, and Stille-type coupling reactions of chloroarenes. J. Am. Chem. Soc..

[16] Mosleh I., Shahsavari H.R., Beitle R., Beyzavi M.H. (2020). Recombinant Peptide Fusion Protein-templated Palladium Nanoparticles for Suzuki-Miyaura and Stille Coupling Reactions. ChemCatChem.

[17] Ghorbani-Choghamarani A., Norouzi M. (2016). Palladium supported on modified magnetic nanoparticles: A phosphine-free and heterogeneous catalyst for Suzuki and Stille reactions. Appl. Organomet. Chem..

[18] Mohazzab B.F., Jaleh B., Issaabadi Z., Nasrollahzadeh M., Varma R.S. (2019). Stainless steel mesh-GO/Pd NPs: Catalytic applications of Suzuki–Miyaura and Stille coupling reactions in eco-friendly media. Green Chem..

[19] Dell’Anna M.M., Lofù A., Mastrorilli P., Mucciante V., Nobile C.F. (2006). Stille coupling reactions catalysed by a polymer supported palladium complex. J. Organomet. Chem..

[20] Yin X., Guo F., Lalancette R.A., Jäkle F. (2016). Luminescent main-chain organoborane polymers: Highly robust, electron-deficient poly (oligothiophene borane) s via Stille coupling polymerization. Macromolecules.

[21] Ryu S.H., Choi S.J., Seon J.H., Jo B., Lee S.M., Kim H.J., Ko Y.-J., Ko K.C., Ahn T.K., Son S.U. (2020). Visible light-driven Suzuki–Miyaura reaction by self-supported Pd nanocatalysts in the formation of Stille coupling-based photoactive microporous organic polymers. Catal. Sci. Technol..

[22] McAfee S.M., McCahill J.S., Macaulay C.M., Hendsbee A.D., Welch G.C. (2015). Utility of a heterogeneous palladium catalyst for the synthesis of a molecular semiconductor via Stille, Suzuki, and direct heteroarylation cross-coupling reactions. RSC Adv..

[23] Saha D., Sen R., Maity T., Koner S. (2013). Anchoring of palladium onto surface of porous metal–organic framework through post-synthesis modification and studies on Suzuki and Stille coupling reactions under heterogeneous condition. Langmuir.

[24] Ghorbani-Choghamarani A., Nikpour F., Ghorbani F., Havasi F. (2015). Anchoring of Pd (II) complex in functionalized MCM-41 as an efficient and recoverable novel nano catalyst in C–C, C–O and C–N coupling reactions using Ph_3_SnCl. RSC Adv..

[25] Khanmoradi M., Nikoorazm M., Ghorbani-Choghamarani A. (2017). Synthesis and characterization of Pd schiff base complex immobilized onto functionalized nanoporous MCM-41 and its catalytic efficacy in the Suzuki, Heck and Stille coupling reactions. Catal. Lett..

[26] Su W., Urgaonkar S., McLaughlin P.A., Verkade J.G. (2004). Highly active palladium catalysts supported by bulky proazaphosphatrane ligands for Stille cross-coupling: Coupling of aryl and vinyl chlorides, room temperature coupling of aryl bromides, coupling of aryl triflates, and synthesis of sterically hindered biaryls. J. Am. Chem. Soc..

[27] Li X., Zhu T., Shao Z., Li Y., Chang H., Gao W., Zhang Y., Wei W. (2016). Newly-generated Al(OH)_3_-supported Pd nanoparticles-catalyzed Stille and Kumada coupling reactions of diazonium salts,(Het) aryl chlorides. Tetrahedron.

[28] Holz J., Pfeffer C., Zuo H., Beierlein D., Richter G., Klemm E., Peters R. (2019). In Situ Generated Gold Nanoparticles on Active Carbon as Reusable Highly Efficient Catalysts for a C–C Stille Coupling. Angew. Chem. Int. Ed..

[29] Ghorbani-Choghamarani A., Naghipour A., Heidarizadi F., Shirkhani R., Notash B. (2016). Bis[(2-methylacetatobenzyl)tri(p-tolyl) phosphonium] hexabromodipalladate (II); Synthesis, characterization, structural study and application as a retrievable heterogeneous catalyst for the amination of aryl halides and Stille cross-coupling reaction. Inorg. Chim. Acta.

[30] Naghipour A., Ghorbani-Choghamarani A., Heidarizadi F., Notash B. (2016). Synthesis, characterization and structural study of a phosphonium salt containing the [Pd_2_Br_6_]_2_−ion and its application as a novel, efficient and renewable heterogeneous catalyst for amination of aryl halides and the Stille cross-coupling reaction. Polyhedron.

[31] Wu W.-Y., Liu L.-J., Chang F.-P., Cheng Y.-L., Tsai F.-Y. (2016). A Highly Efficient and Reusable Palladium (II)/Cationic 2, 2′-Bipyridyl-Catalyzed Stille Coupling in Water. Molecules.

[32] Ogo S., Takebe Y., Uehara K., Yamazaki T., Nakai H., Watanabe Y., Fukuzumi S. (2006). pH-Dependent C−C Coupling Reactions Catalyzed by Water-Soluble Palladacyclic Aqua Catalysts in Water. Organometallics.

[33] Fareghi-Alamdari R., Golestanzadeh M., Bagheri O. (2017). An efficient and recoverable palladium organocatalyst for Suzuki reaction in aqueous media. Appl. Organomet. Chem..

[34] Hao W., Xi Z., Cai M. (2012). A practical synthesis of biaryls and aromatic acetylenes by Stille coupling in room-temperature ionic liquids. Synth. Commun..

[35] Chiappe C., Imperato G., Napolitano E., Pieraccini D. (2004). Ligandless Stille cross-coupling in ionic liquids. Green Chem..

[36] Baran T., Baran N.Y., Menteş A. (2018). An easily recoverable and highly reproducible agar-supported palladium catalyst for Suzuki-Miyaura coupling reactions and reduction of o-nitroaniline. Int. J. Biol. Macromol..

[37] Okamoto K., Akiyama R., Kobayashi S. (2004). Suzuki–Miyaura Coupling Catalyzed by Polymer-Incarcerated Palladium, a Highly Active, Recoverable, and Reusable Pd Catalyst. Org. Lett..

[38] Lin B., Liu Z., Liu M., Pan C., Ding J., Wu H., Cheng J. (2007). Aminophosphine supported on Al_2_O_3_ as recoverable catalyst for the Suzuki coupling. Catal. Commun..

[39] Smith M.D., Stepan A.F., Ramarao C., Brennan P.E., Ley S.V. (2003). Palladium-containing perovskites: Recoverable and reuseable catalysts for Suzuki couplings. Chem. Commun..

[40] Guerra R.R., Martins F.C., Lima C.G., Goncalves R.H., Leite E.R., Pereira-Filho E.R., Schwab R.S. (2017). Factorial design evaluation of the Suzuki cross-coupling reaction using a magnetically recoverable palladium catalyst. Tetrahedron Lett..

[41] Rangraz Y., Nemati F., Elhampour A. (2020). A novel magnetically recoverable palladium nanocatalyst containing organoselenium ligand for the synthesis of biaryls via Suzuki-Miyaura coupling reaction. J. Phys. Chem. Solids.

[42] Lu N., Chen S.-C., Chen T.-C., Liu L.-K. (2008). Palladium-catalyzed Heck reaction under thermomorphic mode. Tetrahedron Lett..

[43] Li C.-K., Ghalwadkar A., Lu N. (2011). Recoverable cationic Pd-catalyzed Heck reaction under thermomorphic mode. J. Organomet. Chem..

[44] Lu N., Lin Y.-C., Chen J.-Y., Fan C.-W., Liu L.-K. (2007). New bis (fluoro-ponytailed) bipyridine ligands for Pd-catalyzed Heck reactions under fluorous biphasic catalysis condition. Tetrahedron.

[45] Lu N., Ou Y.-M., Feng T.-Y., Cheng W.-J., Tu W.-H., Su H.-C., Wang X., Liu L., Hennek M.D., Sayler T.S. (2012). Synthesis and characterization of polyfluorinated 2, 2′-bipyridines and their palladium and platinum complexes [MX_2_(bis(R_f_CH_2_OCH_2_)-2, 2′-bpy)](X = Cl, Br). J. Fluor. Chem..

[46] Lu N., Lin K.Y., Li C.K., Kung C.C., Yeh Y.P., Cheng Y.Y., Liu L.K. (2015). Recyclable palladium catalysts for the Heck/Sonogashira reaction under microwave-assisted thermomorphic conditions. J. Chin. Chem. Soc..

[47] Lu N., Chen Y.-C., Chen W.-S., Chen T.-L., Wu S.-J. (2009). Efficient, Recoverable, Copper-Free Sonogashira Reaction under FBS and Thermomorphic Mode. J. Organomet. Chem..

[48] Lu N., Chen S.C., Lin Y.C., Cheng Y.Y., Liu L.K. (2008). High Fluorine Content Bis (fluoro-Ponytailed) Bipyridine Palladium Complexes as Catalyst for Mizoroki-Heck Reactions under Fluorous Biphasic Catalysis Conditions. J. Chin. Chem. Soc..

[49] Yabe Y., Maegawa T., Monguchi Y., Sajiki H. (2010). Palladium on charcoal-catalyzed ligand-free Stille coupling. Tetrahedron.

[50] Wolf C., Lerebours R. (2003). Efficient Stille cross-coupling reaction using aryl chlorides or bromides in water. J. Org. Chem..

[51] Milstein D., Stille J.K. (1978). A general, selective, and facile method for ketone synthesis from acid chlorides and organotin compounds catalyzed by palladium. J. Am. Chem. Soc..

[52] Lu N., Yeh Y.P., Wang G.B., Feng T.Y., Shih Y.H., Chen D. (2017). Dye-sensitized TiO_2_-catalyzed photodegradation of sulfamethoxazole under blue or yellow light. Environ. Sci. Pollut. Res..

[53] Howell J.L., Lu N., Friesen C.M. (2005). New derivatives of poly-hexafluoropropylene oxide from the corresponding alcohol. J. Fluor. Chem..

[54] Lu N., Tu W.H., Hou H.C., Lin C.T., Li C.K., Liu L.K. (2010). Synthesis, structure and spectroelectrochemical property of (2, 2′-bipyridine)–metal (M = Pt, Pd) dichloride with 4, 4′-bis (fluorous-ponytail) on bipyridine. Polyhedron.

[55] Periyanagounder D., Wei T.C., Li T.Y., Lin C.H., Gonçalves T.P., Fu H.C., Tsai D.S., Ke J.J., Kuo H.W., Huang K.W. (2020). Fast-Response, Highly Air-Stable, and Water-Resistant Organic Photodetectors Based on a Single-Crystal Pt Complex. Adv. Mater..

[56] Lu N., Chen J.Y., Fan C.W., Lin Y.C., Wen Y.S., Liu L.K. (2006). (2, 2′-Bipyridine) palladiumdichloride Derivatives as Recyclable Catalysts in Heck Reactions. J. Chin. Chem. Soc..

[57] Howell J.L., Lu N., Perez E.W., Friesen C.M., Novak I., Waterfeld A., Thrasher J.S. (2004). The preparation of primary poly-hexafluoropropylene oxide halides (poly-HFPO-CF2X where X = I, Br, Cl and F). J. Fluor. Chem..

