# Supplementary material for: Recoverable Palladium-Catalyzed Carbon-Carbon Bond Forming Reactions under Thermomorphic Mode: Stille and Suzuki-Miyaura Reactions
Source: Molecules. 2021 Mar 5;26(5):1414. doi: 10.3390/molecules26051414 (PMC7961810; doi:10.3390/molecules26051414)
Supplement: Supplementary file 1 [file molecules-26-01414-s001.pdf]

Recoverable palladium-catalyzed carbon-carbon bond forming reactions under thermomorphic mode: Stille and Suzuki-Miyaura reactions

Eskedar Tessema [1, †], Vijayanath Elakkat [1,†], Chiao-Fan Chiu [2,3,*], Zong-Lin Tsai [1], Ka Long Chan [1], Chia-Rui Shen[4,5], Han-Chang Su [6] and Norman Lu [1,7,*]

1. Institute of Organic and Polymeric Materials, National Taipei University of Technology, Taipei 106, Taiwan; eskedartessema18@gmail.com (E.T.); vijayanathe123@gmail.com (V.E.); a66366268@gmail.com (Z-L.T.); kalongabc@gmail.com (K.L.C.)
2. Department of Pediatrics, Linkou Medical Center, Chang Gung Memorial Hospital, Taoyuan 333, Taiwan; chioufan2008@gmail.com
3. Graduate Institute of Clinical Medical Sciences, College of Medicine, Chang Gung University, Taoyuan 333, Taiwan
4. Department of Medical Biotechnology and Laboratory Sciences, College of Medicine, Chang Gung Univer-sity, Taoyuan 333, Taiwan; crshen@mail.cgu.edu.tw
5. Department of Ophthalmology, Linkou Medical Center, Chang Gung Memorial Hospital, Taoyuan 333, Taiwan
6. Creditable Service Technology Consultants, New Taipei City 235, Taiwan; henry@mail.creditable.com.tw
7. Development Center for Smart Textile, National Taipei University of Technology, Taipei 106, Taiwan.

Supplementary Materials (SM)

1. **General procedures** **SI2**
2. **Thermomorphic curve** **SI3**
3. **GC/MS data** **SI3**
4. **Monitoring the catalyst 2b by the ^{19}F NMR spectrum** **SI4**

1. General procedures

HP 6890 GC containing a 30 m 0.250 mm HP-1 capillary column with a 0.25 mm stationary phase film thickness was used to censor the reaction. The same GC instrument with a 5973 series mass selective detector was used to Acquire GC/MS data. The flow rate was 1 mL/ min and splitless. NMR spectra were recorded on Bruker AM 500 and Joel AM 200 using 5 mm sample tubes. $CDCl_3$ was the reference for ^{1}H and ^{13}C NMR spectra. Freon® 11 ($CFCl_3$) was the reference for ^{19}F NMR spectra.

2. Thermomorphic curve: plot of solubility versus temperature

Pd complexes **2a-d** and ligand **1e** were dissolved in same amount of deuterated DMF inside the NMR tubes[1]. The $HCF_2(CF_2)_3CH_2OH$, which was totally soluble in DMF in the temperature range of -40 to 80 °C, was used as an internal standard. The variable temperature NMR (^{1}H & ^{19}F) measurements were operated between -40 to 80 °C. The 80 °C was the highest temperature limit for the NMR 500 spectrometer. The general procedures were at the set temperature intervals, after the 256 scans the CF_2 peaks of fluorous compounds, which usually appear around -120 ppm, in ^{19}F NMR spectrum was integrated, and its value was compared to that of the integration of the CF_2 group from standard, $HCF_2(CF_2)_3CH_2OH$. Accordingly, all the ^{1}H NMR spectra results which were also analyzed with similar procedures above were in good agreements with those of ^{1}F NMR spectra. The set temperature point intervals are -40, -20, -10, 0, 20, 30, 40, 50 60, 70, and 80 °C.

3. Coupling products identification

The coupling products are identified by GC/MS data and NMR methods. Their GC/MS data are shown below. These products are all known compounds whose NMR data are the same as the literature data.

Stille products:

4a (R= H) **$C_6H_4CH{=}CH_2$** (m/z; EI): 104 (M^+), 77 (M^+- C_2H_3).

5a (R= CN) **$(CN)C_6H_4CH{=}CH_2$** (m/z; EI): 129 (M^+), 102 (M^+- C_2H_3), 103 (M^+- CN), 77 (M^+- CN-C_2H_3= 77).

6a (R= NO_2) **$(NO_2)C_6H_4CH{=}CH_2$** (m/z; EI): 149 (M^+), 120 (M^+- C_2H_3), 103 (M^+- NO_2), 77 (M^+-NO_2-C_2H_3).

7a (R= CH_3) **$(CH_3)C_6H_4CH{=}CH_2$** (m/z; EI): 118 (M^+), 93 (M^+- C_2H_3), 103 (M^+- CH_3), 77 (M^+- NO_2-C_2H_3).

Suzuki-Miyaura products:

12a (X= Ph, Y= CN) **$(CN)C_6H_4C_6H_4C_6H_5$** (m/z; EI): 255 (M^+), 229 (M^+-CN), 178 (M^+-C_6H_5), 153 (M^+-C_7H_4N).

13a (X= H, Y= CN) **$(CN)C_6H_4C_6H_5$** (m/z; EI): 179 (M^+), 151 (M^+-CN), 102 (M^+-C_6H_5).

13b (X= H, Y= H) **$C_6H_5C_6H_5$** (m/z; EI): 154 (M^+), 77 (M^+-C_6H_5).

13c (X= H, Y= OCH_3) **$(CH_3O)C_6H_4C_6H_5$** (m/z; EI): 184 (M^+), 153 (M^+-OCH_3), 107 (M^+-C_6H_5).

4. **Monitoring** the catalyst **2b** by the ^{19}F NMR spectrum.

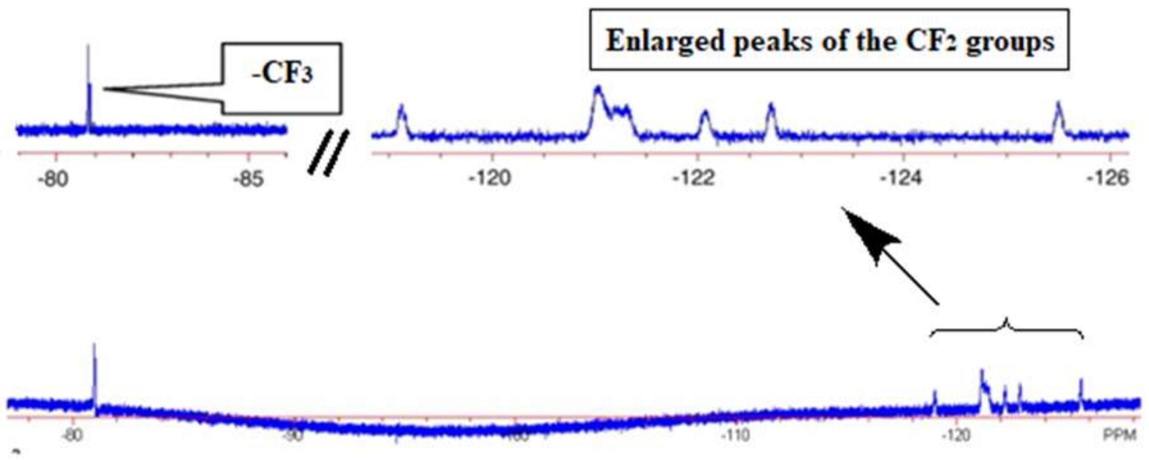

Figure S1. The comparison of ^{19}F NMR spectra of catalyst **2b** from Pd-catalyzed Suzuki-Miyaura reaction (**top image**) before the reaction; (**bottom image**) after the 8 cycles.

^{19}F NMR data of **2b** (NMR data collected in d-DMF at 90 C because of poor solubility.)[1, 2]:
^{19}F NMR (470.5 MHz, DMF-d6, d ppm, J Hz) 80.9 (t, 6F, ^{3} J_{FF} = 7.52, –CF_3), CF_2 groups region: 121.0 (4F), 121.3 (20F), 122.1 (4F), 122.7 (4F), 125.5 (4F).

Reference:

1. Lu, N.; Chen, S.-C.; Chen, T.-C.; Liu, L.-K. Palladium-catalyzed Heck reaction under thermomorphic mode. *Tetrahedron Letters* **2008**, *49*, 371-375.
2. Lu, N.; Chen, Y.-C.; Chen, W.-S.; Chen, T.-L.; Wu, S.-J. Efficient, Recoverable, Copper-Free Sonogashira Reaction under FBS and Thermomorphic Mode. *J. Organomet. Chem.* **2009**, *694*, 278-284.
